# Clinical and Pathological Features of Breast Cancer in Systemic Sclerosis: Results from the Sclero-Breast Study

**DOI:** 10.3390/jpm11060580

**Published:** 2021-06-20

**Authors:** Angela Toss, Amelia Spinella, Chrystel Isca, Caterina Vacchi, Guido Ficarra, Luca Fabbiani, Anna Iannone, Luca Magnani, Paola Castrignanò, Pierluca Macripò, Elisa Gasparini, Simonetta Piana, Laura Cortesi, Antonino Maiorana, Carlo Salvarani, Massimo Dominici, Dilia Giuggioli

**Affiliations:** 1 Department of Oncology and Hematology, Azienda Ospedaliero-Universitaria di Modena, 41124 Modena, Italy; isca.chrystel@gmail.com (C.I.); hbc@unimore.it (L.C.); massimo.dominici@unimore.it (M.D.); 2 Department of Surgery, Medicine, Dentistry and Morphological Sciences with Transplant Surgery, Oncology and Regenerative Medicine Relevance, University of Modena and Reggio Emilia, 41124 Modena, Italy; anna.iannone@unimore.it; 3 SSc Unit, Rheumatology Unit, Azienda Ospedaliero-Universitaria di Modena, 41124 Modena, Italy; amelia.spinella@gmail.com (A.S.); vacchi.caterina@outlook.it (C.V.); castrignanopaola@gmail.com (P.C.); pier.macripo@gmail.com (P.M.); carlo.salvarani@unimore.it (C.S.); dilia.giuggioli@unimore.it (D.G.); 4 Pathology Unit, University Hospital of Modena, 41124 Modena, Italy; ficarra.guido@aou.mo.it (G.F.); luca.fabbiani@unimore.it (L.F.); antonino.maiorana@unimore.it (A.M.); 5 Department of Medical and Surgical Sciences for Children and Adults, University of Modena and Reggio Emilia, 41124 Modena, Italy; 6 Rheumatology Unit, Azienda Unità Sanitaria Locale (AUSL)-IRCCS di Reggio Emilia, 42123 Reggio Emilia, Italy; magnani.l@gmail.com; 7 Oncology Unit, Azienda Unità Sanitaria Locale (AUSL)-IRCCS di Reggio Emilia, 42123 Reggio Emilia, Italy; elisa.gasparini2@ausl.re.it; 8 Pathology Unit, Azienda Unità Sanitaria Locale (AUSL)-IRCCS di Reggio Emilia, 42123 Reggio Emilia, Italy; simonetta.piana@ausl.re.it

**Keywords:** breast cancer, systemic sclerosis, clinical stage, interstitial lung disease, pulmonary arterial hypertension, cancer screening

## Abstract

Systemic Sclerosis (SSc) is a chronic disease associated with a 1.5-fold increase in cancer risk, including lung cancer, hematological malignancies, and breast cancer (BC). This is a retrospective study aiming to explore the clinical and pathological features of BC developed by SSc patients. A total of 54.5% of patients developed BC before SSc (median interval: 5 years), whereas 45.5% of patients developed BC after SSc (median delay: 8 years). A total of 93.1% of patients were diagnosed with an early stage tumor. Among invasive carcinomas, 70.8% presented with a low Mib1, 8.3% with a tubular histotype, and 42.8% with a Luminal A-like phenotype. A total of 66.6% of patients underwent breast-conserving surgery and 55.5% RT. A total of 40% of patients developed interstitial lung disease after RT and 20% diffuse cutaneous SSc. The cause of death of the six deceased patients was PAH. A significant association was observed between the use of immunosuppressive therapy and diffuse skin extension, negative ACA, positive Anti-Scl-70, and interstitial lung disease, but not BC status. SSc patients developed BC at a good prognosis, suggesting a de-escalation strategy of cancer therapies. In particular, ionizing radiation and chemotherapeuticals should be limited to higher-risk cases. Finally, proper screening is mandatory in order to allow for early cancer detection in SSc patients.

## 1. Introduction

Systemic Sclerosis (SSc) is a chronic disease characterized by vascular dysfunction, specific autoimmune alterations, and fibrosis of the skin and internal organs, such as lungs and heart. However, gastrointestinal and kidney involvement is also well known [1,2]. Previous studies have shown a 1.5-fold increase in cancer risk in SSc patients compared with the general population, especially lung cancer, hematological malignancies, and breast cancer (BC) [3,4,5,6,7,8,9]. Many theories have been put forward to explain this association: firstly, exposure to immunosuppressive therapies; secondly, genetic susceptibility to both malignancy and the development of autoimmune disease; and finally, a common inciting exposure or more likely a multifactorial pathogenic mechanism involving both genetic contributions as well as other cancer risk factors [10,11,12,13].

The relationship between BC and SSc has long been debated. Previous research has been contradictory and inconclusive on this matter, and breast is one of the organs not directly involved by the tissue alterations that characterize SSc. Overall, several authors described the association between SSc and BC [6,14,15,16], while the standardized incidence ratio of BC in female SSc patients was found to be 1.62 (95% confidence intervals: 0.7–3.19) [17]. In 2006, Scope et al. retrospectively analyzed clinical data of 65 SSc patients affected by BC, reporting that 75% of scleroderma and BC cases were diagnosed within 3 years of each other and in 44% of cases the scleroderma appeared before or simultaneously with the diagnosis of BC [14]. In 2007, Derk et al. found that SSc patients affected by BC presented pulmonary fibrosis more frequently than the other patients without malignancy [10]. In 2008, Lu et al. described 21 cases of BC in SSc patients and analyzed their exposure to BC risk factors through standardized self-administered questionnaires and case note reviews. In this analysis, scleroderma patients with BC were found to have a higher incidence of a positive family history of BC and a lower incidence of hormone-replacement therapy use [11]. In 2012, Hashimoto et al. studied 405 SSc patients and identified four cases of BC, indicating heart involvement of SSc as a significant risk factor for BC [12]. In 2014, Colaci et al. observed a significant increase in BC incidence compared with the general population from the same geographic area with a close temporal relationship between SSc and BC onset [6]. This close temporal relationship suggests that early onset of BC might trigger the immune system, leading to an excessive immune response and the onset of SSc [13].

To our knowledge, nevertheless, no study has evaluated the clinical and pathological features of BC developed by SSc patients and their correlations with SSc features. The aim of our project was to analyze clinical and pathological features of BC in 33 SSc patients attending two Rheumatology/SSc Units in the north of Italy.

## 2. Materials and Methods

This is an observational retrospective multicenter study performed at Modena University Hospital and Reggio Emilia Hospital in northern Italy. We enrolled women affected by both SSc and BC in their lifespan, regardless of which disease was developed first. Women affected by SSc with a personal history of BC were identified at two Rheumatology/SSc Units in the north of Italy between January 2017 and December 2019. Clinical and pathological characteristics of BC were evaluated in terms of particular age at diagnosis, menopausal status, histotype, estrogen receptor status (ER), progesterone receptor status (PR), Mib1 and HER2 expression, clinical and pathological stage at diagnosis, metastatic sites, type of loco-regional treatment (surgery and radiotherapy), type of systemic treatment (neoadjuvant/adjuvant chemotherapy and endocrine treatment), other cancers, and time from the diagnosis of the first disorder to the second one. 

Moreover, clinical–rheumatological features related to SSc were collected: age at disease onset; smoking habits (with packs/year); skin extension of the disease (limited/diffuse); presence of skin ulcers, calcinosis, and telangiectasia; presence of gastro-intestinal and kidney involvement; interstitial lung disease (at HR-CT); forced vital capacity (FVC) evaluation at pulmonary function tests and diffusing capacity of the lungs for carbon monoxide through the single-breath technique (DLCO SB); ECG abnormalities; echocardiographic assessment of pulmonary arterial hypertension (PAH) with measurement of pulmonary artery systolic pressure (PAPs); SSc pattern at videocapillaroscopy; autoantibody profile; exposure to immunosuppressive and vasoactive therapies; status at last follow-up evaluation; and cause of death. Oncological and rheumatological characteristics were then compared with data available in the literature for each disease.

ER, PR, and HER2 expression were determined according to the national pathology guidelines, which closely adhere to international standards. ER and PR were defined as positive with immunohistochemical staining of at least 1% of nuclei. Triple negativity was defined as immunohistochemical staining of less than 1% of nuclei for both ER and PR, and an immunohistochemical result (DAKO score) of 0 or 1+ for HER2/neu.

Statistical analysis was performed using SPSS (IBM Software, New York, NY, USA, version 22.0). Fisher’s exact test was applied. *p* < 0.05 was considered statistically significant. 

Approval for this study was obtained from the local ‘Area Vasta Emilia Nord (AVEN)’ Ethics Committee (practice n.376/17, protocol n.4442/EC) and written informed consent was obtained from all subjects included in the study.

## 3. Results

### 3.1. Clinical–Pathological Features of Breast Cancer

Between January 2017 and December 2019, 33 patients affected by SSc with a personal history of BC were identified. Clinical–pathological characteristics of the overall population are listed in Table 1. Median age at BC diagnosis was 58 years (range 43–75), with 8 (26.7%) premenopausal women and 22 (73.3%) post-menopausal patients (3 unknown). At histological examination (biopsy samples or surgical specimens in stage I–III BC and biopsy samples in stage IV BC), 4 of 28 patients (14.3%) developed in situ carcinoma (4 DCIS), while 24 (85.7%) were categorized as invasive carcinomas (79.2% ductal, 12.5% lobular, and 8.3% tubular carcinoma). Overall, 75% of invasive BCs were ER positive, 70.8% were PR positive, 70.8% showed Mib1 ≤ 20%, and 28.6% over-expressed HER2. As for molecular subtype, nine patients (42.8%) presented with Luminal A-like BC, two (9.5%) with Luminal B-like HER2-negative BC, five (23.8%) with Luminal B-like HER2-positive BC, one (4.8%) with HER2 enriched-like BC, and four (19%) with triple-negative BC. Nineteen patients (65.5%) were diagnosed with a clinical stage I BC and 8 women (27.6%) with stage II tumors. With respect to pathological stage, 5 patients (17.2%) showed stage 0 (in situ) BC, 15 patients (51.8%) stage I disease, and 7 patients (24.1%) stage II BC. Only one patient was diagnosed with de novo stage IV BC. Concerning local cancer treatments, 20 patients (66.6%) underwent breast-conserving surgery and 15 patients (55.5%) underwent RT after surgery. Moreover, 8 of the 12 patients (66.6%) that did not undergo RT were already affected by SSc at the time of BC diagnosis. With regard to systemic treatments, 3 patients (10.3%) underwent neoadjuvant chemotherapy (with no pathologic complete response), 11 patients (37.9%) adjuvant chemotherapy, and 15 (62.5%) were prescribed endocrine therapies. Two patients relapsed during follow up. Eight patients developed other cancers: five skin cancers, one lymphoma, one lung cancer, and one contralateral BC.

### 3.2. Rheumatological SSc Features

The rheumatological characteristics of the overall SSc population are listed in Table 2. The median age at SSc onset was 57 years (range 32–73). Eighteen patients (54.5%) developed BC before SSc with a median time between the two diagnoses of 5 years, whereas 15 patients (45.5%) developed BC after their SSc diagnosis with a median delay of 8 years. Six patients (18.7%) were smokers or ex-smokers and most of them smoked 20 packs per year. In 23 cases (71.9%), patients presented with limited skin extension (lc-SSc), 13 patients (39.4%) showed skin ulcers, 8 patients (24.2%) calcinosis, and 24 patients (72.7%) telangiectasia. Gastro-intestinal involvement was present in 18 patients (54.5%), whereas kidney involvement was present in no one and interstitial lung disease at HRCT in 18 patients (54.5%). Six of the 15 patients (40%) who underwent radiation therapy developed interstitial lung disease at HRCT, whereas three patients (20%) developed diffuse cutaneous scleroderma (dc-SSc). Concerning autoantibody profile, ANA test was positive in 21 cases (67.7%); specifically, ACA in 14 (45.1%) cases, ANoA in 3 (9.7%) cases, and Anti-Scl-70 in 9 (29%) cases. PAPs was >35 mmHg in 12 cases (37.5%), FVC < 75% in 6 cases (18.2%), and DLCO SB < 70% in 22 cases (73.3%). ECG disorders were described in three patients (9.1%), with atrial fibrillation for two patients and atrial flutter for one patient. Evaluation at capillaroscopy was observed to be abnormal in 21 patients (67.7%), with an active SSc pattern in 11 patients (33.3%). Immunosuppressive therapy was prescribed in five cases (15.6%); in particular, cyclophosphamide (CFX) and mycophenolate mofetil (MMF) for two cases each, methotrexate (MTX) for one case, and other drugs for three cases. A significant association between the use of immunosuppressive therapy (yes vs. no) and diffuse skin extension (*p* = 0.015), negative ACA status (*p* = 0.036), positive Scl70 status (*p* = 0.017), and presence of interstitial lung disease at HR-CT (*p* = 0.036) was observed. Vasoactive agents were used to treat 25 patients (75.7%); in particular, iloprost in 18 patients, calcium channel blockers (CCBs) in 12 patients, bosentan in 3 patients, and sildenafil in 2 patients.

At the end of December 2019, 22 patients (66.7%) were recorded as still alive, with 5 patients (15.1%) lost at follow up. In all six deceased patients, the cause of death was PAH (in one case with concomitant lung cancer).

Positive ER status of BC was significantly associated with a normal SSc pattern at videocapillaroscopy (*p* = 0.030). No other statistically significant associations were observed between BC pathological stage, Mib1 level or ER expression, and skin extension of the disease, the presence of skin ulcers, calcinosis, teleangectasia, gastro-intestinal and kidney involvement, interstitial lung disease (at HR-CT), FVC and DLCO SB values, echocardiographic assessment of PAH with PAPs measurement, ECG abnormalities, SSc pattern at videocapillaroscopy, and autoantibody profile (ANA, ACA, ANoA, and Anti-Scl-70) (Table 3).

## 4. Discussion

Several authors have described the association between SSc and BC [6,14,15,16], while the standardized incidence ratio of BC in female SSc patients was found to be 1.62 (95% confidence intervals: 0.7–3.19) [17]. To our knowledge, nevertheless, no study has evaluated the clinical and pathological features of BC developed by SSc patients and their correlations with SSc features. Our retrospective multicenter study analyzed clinical and pathological features of BC in 33 SSc patients attending two Rheumatology/SSc Units in the north of Italy.

Overall, 54.5% of these patients developed BC before SSc with a median time between the two diagnoses of 5 years, whereas 45.5% of patients developed BC after their SSc diagnosis with a median delay of 8 years. Our findings diverge from the previous studies that showed a close temporal relationship between SSc and BC onset. In particular, in a retrospective cohort of 65 SSc patients affected by BC, Scope et al. reported that 75% of scleroderma and BC cases were diagnosed within 3 years of each other and in 44% of cases, scleroderma appeared before or simultaneously with the BC diagnosis [16]. Moreover, our group previously observed a significant increase in BC incidence compared with the general population from the same geographic area, with a median 2.5-year interval between SSc onset and the cancer [6]. This delayed temporal relationship suggests that early BC onset might not be the trigger for the immune system to lead to SSc onset. In addition, it indicates that SSc might not be a paraneoplastic manifestation anticipating BC, as previously hypothesized [9,13]. Therefore, many factors should be considered in the common pathogenesis of these two disorders [18]. First of all, the female susceptibility observed for SSc suggests an influence of the same hormonal factors found to be involved in BC, such as elevated prolactin levels and decreased levels of dehydroepiandrosterone sulfate (DHEA) [19,20]. Secondly, calcium channel blockers (CCBs), a cornerstone treatment for SSc vasculopathy, have been suspected to be a risk factor for BC in the general population [21,22,23,24]. Lastly, several immunosuppressive drugs can be used in SSc but may contribute to cancer [25,26], whereas several chemotherapeutic agents (such as taxanes) and ionizing radiations have been associated with tissue fibrosis and/or scleroderma and may exacerbate pre-existing systemic scleroderma [27,28,29,30,31].

In line with data from the general population, 75% of invasive tumors were hormone receptor positive, 28.6% were HER2 positive, and 19% were triple-negative. Nevertheless, it is worth noting that 93.1% of BC patients were diagnosed with a clinical stage I or II tumor. Among invasive carcinomas, 70.8% presented with a low Mib1 proliferative rate, and 8.3% with a special histotype at a very good prognosis (tubular), while 42.8% presented with a Luminal A-like phenotype. According to such good prognostic features, only 10.3% of patients underwent neoadjuvant chemotherapy, 37.9% adjuvant chemotherapy, 66.6% breast-conserving surgery, and 55.5% underwent RT after surgery. Interestingly, 40% of SSc patients that underwent RT developed interstitial lung disease and 20% diffuse cutaneous forms of SSc.

As is well known, pulmonary involvement is one of the most important features of SSc and often the leading cause of exitus. Interstitial lung disease and pulmonary hypertension together account for 60% of SSc-related deaths [32,33]. Additionally, heart involvement is clinically detectable in about 30–50% of cases at large [34] and produces several clinical patterns, including hemodynamic, electric, and vascular disorders. Cardiovascular and pulmonary involvement, alone or in association, were confirmed to be the most frequent complications (54.5%) affecting overall disease outcome in a large study on SSc survival and disease pathomorphosis [35]. In line with all these data, 54.5% of the BC patients in our study presented with interstitial lung disease. In all six deceased patients, furthermore, the cause of death was PAH.

In 2007, Derk et al. found that SSc patients affected by BC presented with pulmonary fibrosis more frequently than other patients without malignancy [10]. In 2012, moreover, Hashimoto et al. indicated heart involvement of SSc as a significant risk factor for BC [12]. Given the limit of our small sample size, nevertheless, the impact of cardio-pulmonary impairment in our BC SSc patients was comparable with the main literature on this topic in the general population of SSc patients. A detailed analysis with quantitative HRCT fibrosis score for ILD and right heart catheterization for PAH might be more sophisticated and useful to refine overall data. For this reason, this will be the subject of our future research.

In a recent cohort analysis on cardio-pulmonary involvement in SSc [36], we interestingly found a different immune pattern with a higher prevalence of Anti-Scl-70 antibodies in patients with dc-SSc and a higher prevalence of ACA antibodies in patients with lc-SSc. It is worth noting that patients with ACA antibodies showed greater cardio-pulmonary involvement in terms of PAH, whereas an overall worse prognosis was associated with Anti-Scl-70 in previous studies [34]. Other authors studied possible correlations between autoantibody assessment and BC cancer risk [18,37,38,39,40,41]. On the whole, based on these observations, a novel concept of autoimmunity as a response to an underlying malignancy has been advanced for SSc and may contribute to new strategies in patient management. Indeed, autoantibodies could be useful biomarkers for screening strategies, as proposed by some of these authors. Even if standardized effectiveness and usefulness remain to be demonstrated, a strategy based on repeated and more aggressive screening in patients with specific autoantibody subsets or in seronegative patients may be tempting. On the grounds of our experience with BC SSc patients, we found a significant association between the use of immunosuppressive therapy and diffuse skin extension, negative ACA, positive Anti-Scl-70 assessment, and interstitial lung disease at HR-CT, but no correlations with BC status were observed.

Possibly due to the small sample size, besides positive ER status and normal SSc pattern at videocapillaroscopy, no statistically significant associations were observed between any oncological characteristic (pathological stage, Mib1 level, or ER expression) and rheumatological features such as skin extension of the disease, presence of skin ulcers, calcinosis, teleangectasia, and gastro-intestinal and kidney involvement. Similarly, interstitial lung disease at HR-CT, pulmonary function test values, echo assessment of PAH, ECG abnormalities, SSc videocapillaroscopic pattern, and autoantibody status revealed no significant correlations with the BC characteristics mentioned above.

## 5. Conclusions

Overall, confirming previous research in systemic rheumatic diseases [42], the SSc patients in our study developed BC with a good prognosis (mostly early diagnosis and a Luminal-like HER2-negative biology). Along with data already available in the literature on adverse events of oncological treatments in SSc patients, our findings might suggest a de-escalation strategy of cancer therapies in this setting. In particular, in order to balance benefits and harms in this fragile subset of patients, ionizing radiation exposure and chemotherapy, such as taxanes, might be limited to higher-risk cases. Furthermore, the risk of second cancers (mostly myelodysplastic syndrome and acute myelogenous leukemia) after alkylating agents, platinum-based drugs, and anthracycline should be taken into account in the selection of oncological therapies in those patients at increased cancer risk.

On these grounds, proper screening of SSc patients is mandatory in order to allow for early detection of tumor development. Further investigations on larger numbers of patients are needed, yielding substantial benefits at various levels. First of all, they would further clarify the intriguing relationship between BC and SSc. Secondly, they would help us explore the common biological and molecular pathways at the basis of SSc and BC development, with the aim of improving BC diagnosis and prognostication and personalizing oncological targeted treatments in this specific subset of multimorbid patients.

## Figures and Tables

**Table 1:** Clinical–pathological characteristics of breast cancer (BC) developed by patients in the study population.

	Overall Population, 33 Patients n (%)
Median age at BC diagnosis:	58 years (43–75)
Menopausal status:	
Premenopause	8 (26.7%)
Postmenopause	22 (73.3%)
Unknown	3
Histological Examination:	
In situ carcinoma	4 (14.3%)
DCIS	4 (100%)
Invasive carcinoma:	24 (85.7%)
Ductal	19 (79.2%)
Lobular	3 (12.5%)
Tubular	2 (8.3%)
Unknown	5
IHC characteristics of invasive BC:	
ER STATUS:	
ER positive	18 (75%)
ER negative	6 (25%)
PR STATUS:	
PR positive	17 (70.8%)
PR negative	7 (29.2%)
Mib1:	
Mib1 ≤ 20%	17 (70.8%)
Mib1 > 20%	7 (29.2%)
HER2 EXPRESSION:	
0	9 (42.8%)
1+	5 (23.8%)
2+ (ISH not amplified)	1 (4.8%)
2+ (ISH amplified)	1 (4.8%)
3+	5 (23.8%)
Unknown	3
Molecular Subtype of invasive BC:	
Luminal A-like	9 (42.8%)
Luminal B-like/HER2 negative	2 (9.5%)
Luminal B-like/HER2 positive	5 (23.8%)
HER2 enriched-like	1 (4.8%)
Triple negative	4 (19%)
unknown	3
Clinical Stage:	
I	19 (65.5%)
II	8 (27.6%)
III	1 (3.4%)
IV	1 (3.4%)
Unknown	4
Pathological Stage:	
0 (in situ)	5 (17.2%)
I	15 (51.8%)
II	7 (24.1%)
III	1 (3.4%)
IV	1 (3.4%)
Unknown	4
Type of surgery:	
Conserving surgery	20 (66.6%)
Mastetctomy	9 (30%)
Not done	1 (3.3%)
Unknown	3
Radiotherapy:	
Yes	15 (55.5%)
No	12 (44.4%)
Unknown	6
Neoadjuvant chemotherapy:	
Anthracycline + Taxane	1 (3.4%)
Taxane + trastuzumab + pertuzumab	1 (3.4%)
Anthracycline + taxane + trastuzumab	1 (3.4%)
Not done	26 (89.7%)
Unknown	4
Pathologic Complete Response (pCR):	
yes	0
no	3 (100%)
Adjuvant chemotherapy:	
Anthracycline	3 (10.3%)
anthra + taxane	2 (6.9%)
Anthra + taxane + trastuzumab	3 (10.3%)
anthra + trastuzumab + pertuzumab	1 (3.4%)
Anthra + trastuzumab	1(3.4%)
CMF	1 (3.4%)
Not done	18 (62%)
Unknown	4
Endocrine treatment:	
Tamoxifene	4 (16.7%)
NSAI	9 (37.5%)
Tam switch to NSAI or exemestane	2 (8.3%)
Not done	9 (37.5%)
Unknown or in situ BC	9
Metastatic sites (1 stage IV de novo, 2 relapses):	
Visceral	1 (50%)
Visceral + bone	1 (50%)
None	27 (93%)
Unknown	4
Other cancers:	
Basal cell carcinoma	4 (50%)
Squamous cell carcinoma of the skin	1 (12.5%)
Marginal zone lymphoma	1 (12.5%)
Lung cancer	1 (12.5%)
Breast Cancer	1 (12.5%)

**Table 2:** Clinical–pathological characteristics of systemic sclerosis (SSc) developed by patients in the study population.

	Overall Population, 33 Patients n (%)
Age at SS onset:	57 years (32–73)
Smoking Habits:	
Non-smoker	26 (81.2%)
Ex-smoker	5 (15.6%)
Smoker	1 (3.1%)
Unknown	1
Packs-year:	
0	25 (80.6%)
10	1 (3.2%)
20	3 (9.7%)
30	1 (3.2%)
>100	1 (3.2%)
Unknown	2
Skin extension:	
limited lc-SSc	23 (71.9%)
diffuse dc-SSc	9 (28.1%)
Unknown	1
Skin Ulcers:	
yes	13 (39.4%)
no	20 (60.6%)
Calcinosis:	
yes	8 (24.2%)
no	25 (75.7%)
Telangiectasia:	
yes	24 (72.7%)
no	9 (27.3%)
Gastro-intestinal involvement:	
yes	18 (54.5%)
no	15 (45.4%)
Kidney involvement:	
yes	0
no	33 (100%)
Interstitial lung disease (HRCT):	
yes	18 (54.5%)
no	15 (45.4%)
ANA:	
yes	21 (67.7%)
no	10 (32.2%)
Unknown	2
ACA:	
yes	14 (45.1%)
no	17 (54.8%)
unknown	2
ANoA:	
yes	3 (9.7%)
no	28 (90.3%)
unknown	2
Anti-Scl-70:	
yes	9 (29%)
no	22 (71%)
unknown	2
PAPs > 35 mmHg:	
yes	12 (37.5%)
no	20 (62.5%)
unknown	1
FVC < 75%:	
yes	6 (18.2%)
no	26 (78.8%)
unknown	1 (3%)
DLCO SB:	
<70%	22 (73.3%)
≥70%	8 (26.6%)
unknown	3
ECG disorders:	
none	30 (90.9%)
FA	2 (6.1%)
Atrial Flutter	1 (3%)
Capillaroscopy pattern:	
Normal	10 (30.3%)
Early	3 (9.1%)
Active	11 (33.3%)
Late	7 (21.2%)
unknown	2 (6%)
Immunosuppressive therapy:	
Any	5 (15.6%)
None	27 (84.4%)
unknown	1
Cyclophosphamide (CFX)	2 (6.2%)
Mycophenolate mofetil (MMF)	2 (6.2%)
Methotrexate (MTX)	1 (3.1%)
Others (Azathioprine, Hydroxychloroquine, unknown)	3 (9.3%)
Vasoactive therapy:	
Any	25 (75.7%)
None	8 (24.2%)
Iloprost	18 (54.5%)
Calcium channel blockers (CCBs)	12 (36.4%)
Bosentan	3 (9.1%)
Sildenafil	2 (6.1%)
Status at last follow up:	
Dead	6 (18.2%)
Alive	22 (66.7%)
Lost	5 (15.1%)
Cause of death:	
Pulmonary arterial hypertension (PAH)	5 (83.3%)
Pulmonary arterial hypertension and lung cancer	1 (16.6%)

**Table 3:** Associations within clinical–pathological characteristics of BC and SSc.

	Immunosuppressive Therapy (IT) (Yes, No)	BC Characteristics			
		BC Pathological Stage (In Situ/Invasive)	BC Pathological Stage (I + II, III + IV)	Mib1 (≤20%, >20%)	ER Status (Positive, Negative)
Skin extension (limited/diffuse)	diffuse extension/yes IT *p* = 0.015	*p* = 0.448	*p* = 0.286	*p* = 0.392	*p* = 0.490
Skin ulcers (yes, no)	*p* = 0.669	*p* = 0.266	*p* = 0.623	*p* = 0.302	*p* = 0.319
Calcinosis (yes, no)	*p* = 0.650	*p* = 0.347	*p* = 0.569	*p* = 0.202	*p* = 0.634
Teleangectasia (yes, no)	*p* = 0.582	*p* = 0.425	*p* = 0.069	*p* = 0.480	*p* = 0.520
Gastro-intestinal involvement (yes, no)	*p* = 0.591	*p* = 0.671	*p* = 0.335	*p* = 0.238	*p* = 0.397
Interstitial lung disease (at HR-CT) (yes, no)	yes lung disease/yes IT *p* = 0.036	*p* = 0.604	*p* = 0.296	*p* = 0.320	*p* = 0.320
FVC < 75% (yes, no)	*p* = 0.228	*p* = 0.715	*p* = 0.214	*p* = 0.709	*p* = 0.608
DLCO SB values (<70%, ≥70%)	*p* = 0.621	*p* = 0.731	*p* = 0.741	*p* = 0.184	*p* = 0.123
PAPs >35 mmHg (yes, no)	*p* = 0.634	*p* = 0.417	*p* = 0.683	*p* = 0.680	*p* = 0.320
ECG abnormalities (none, FA + atrial flutter)	*p* = 0.400	*p* = 0.554	*p* = 0.800	*p* = 0.403	*p* = 0.403
SSc pattern at videocapillaroscopy (normal, early + active + late)	*p* = 0.472	*p* = 0.523	*p* = 0.613	*p* = 0.208	normal/positive *p* = 0.030
ANA (yes, no)	*p* = 0.120	*p* = 0.629	*p* = 0.630	*p* = 0.380	*p* = 0.631
ACA (yes, no)	no ACA/yes IT *p* = 0.036	*p* = 0.240	*p* = 0.556	*p* = 0.594	*p* = 0.583
ANoA (yes, no)	*p* = 0.422	*p* = 0.079	*p* = 0.889	*p* = 0.773	*p* = 0.727
Anti-Scl-70 (yes, no)	yes Scl-70/yes IT *p* = 0.017	*p* = 0.472	*p* = 0.296	*p* = 0.160	*p* = 0.651

## Data Availability

The data that support the findings of this study are available from the corresponding author, A.T., upon reasonable request.
